# Perceptual Decision Advantages in Open-Skill Athletes Emerge near the Threshold of Awareness: Behavioral, Computational, and Electrophysiological Evidence

**DOI:** 10.3390/brainsci16020198

**Published:** 2026-02-07

**Authors:** Xudong Liu, Shiying Gao, Yanglan Yu, Anmin Li

**Affiliations:** 1 School of Psychology, Shanghai University of Sport, Shanghai 200438, China; liuxudong202020@163.com (X.L.);; 2 Center for Exercise and Brain Science, Shanghai University of Sport, Shanghai 200438, China

**Keywords:** perceptual decision-making, near-threshold perception, open-skill athletes, evidence accumulation, drift-diffusion model, event-related potentials (ERP)

## Abstract

**Highlights:**

**What are the main findings?**

- Open-skill athletes show perceptual decision advantages selectively near the threshold of awareness.
- These advantages are driven by higher evidence accumulation rates (drift rate) rather than response strategy or non-decision time.

**What are the implications of the main findings?**

- Early sensory ERP components (N2, P2) are similarly modulated by stimulus visibility in athletes and non-athletes.
- Enhanced and earlier P3 differentiation, as well as stronger P3–behavior coupling, indicate more efficient decision-related processing in athletes.

**Abstract:**

Background/Objectives: Perceptual awareness and decision formation unfold gradually as sensory evidence increases. Near the threshold of awareness, small differences in neural processing efficiency can be amplified into marked behavioral variability. Open-skill athletes are trained to make rapid decisions under dynamic and uncertain conditions, yet it remains unclear whether their perceptual advantage reflects enhanced early sensory sensitivity or more efficient late-stage evidence accumulation. This study aimed to identify the processing stage at which open-skill sports expertise exerts its influence. Methods: Twenty-five open-skill athletes and twenty-three non-athlete controls completed a visual orientation discrimination task with eight graded levels of stimulus visibility, ranging from subliminal to clearly visible. Behavioral performance was analyzed together with hierarchical drift–diffusion modeling to estimate latent decision parameters. Event-related potentials (ERPs) were recorded using a 64-channel EEG system during an active decision task and a passive viewing task, focusing on early (N2, P2) and late (P3) components. ERP–behavior correlations were examined across visibility levels. Results: No group differences were observed at the lowest visibility levels. Group differences emerged selectively at intermediate to high visibility levels, where athletes showed higher accuracy and a tendency toward faster responses. Drift–diffusion modeling revealed that this advantage was driven by higher drift rates in athletes, with no group differences in non-decision time, boundary separation, or starting point. Early ERP components (N2, P2) were strongly modulated by stimulus visibility but showed no consistent group differences. In contrast, the P3 component exhibited earlier and more pronounced differentiation across visibility levels in athletes. In the passive viewing task, group differences were substantially reduced. ERP–behavior analyses showed stronger and earlier P3–behavior coupling in athletes. Conclusions: Open-skill sports expertise selectively optimizes late-stage evidence accumulation and its translation into behavior, rather than enhancing unconscious or early sensory processing.

## 1. Introduction

Visual perception is not an all-or-none process but changes continuously as the strength of sensory evidence varies [1,2,3]. When stimulus strength approaches the threshold of awareness, the perceptual system must integrate and evaluate weak sensory signals under conditions of high uncertainty. This stage is therefore considered a critical window in perceptual decision-making, where individual differences are most likely to be amplified [4,5,6,7]. Recent studies using graded stimulus strength have further demonstrated that near-threshold conditions play a central role in understanding conscious access, evidence accumulation, and decision formation [8,9]. Recent electrophysiological work has further shown that late ERP components, particularly the P3, are closely linked to evidence accumulation and decision formation processes during perceptual decisions [10].

Against this background, sport expertise, particularly in open-skill sports, has been proposed to shape more efficient perceptual decision-making mechanisms. Athletes engaged in open-skill sports are repeatedly exposed to dynamic, rapidly changing, and highly uncertain environments, requiring them to integrate multiple sources of sensory information and generate accurate responses within limited time [11,12,13,14]. Such experience has been hypothesized to optimize the transformation of sensory evidence into decisional output [15,16]. However, it remains unclear whether this advantage is expressed uniformly across all levels of perceptual processing or whether it emerges specifically when sensory evidence becomes just sufficient to support reliable decisions.

Behavioral studies have shown that athletes often outperform non-athletes in tasks involving visual search, motion prediction, and rapid responses [11,12]. However, these studies typically employ high-visibility or clearly discriminable stimuli, making it difficult to determine whether sport expertise enhances early sensory sensitivity or primarily optimizes later decisional computations [17]. At the same time, research on visual awareness suggests that under fully subliminal conditions, both behavioral and neural measures tend to show limited and unstable individual differences, whereas near the perceptual threshold, subtle differences in neural processing efficiency are more likely to translate into observable behavioral variability [1,5].

Accordingly, an unresolved question is whether the perceptual decision advantage associated with open-skill sport expertise is selectively expressed at near-threshold levels of awareness, rather than reflecting a general enhancement of unconscious or subliminal processing. Addressing this question is essential for clarifying the processing stage at which sport training exerts its influence and for constraining computational accounts of perceptual decision-making.

To this end, the present study systematically manipulated stimulus visibility across a continuous range and compared open-skill athletes and non-athletes in a perceptual decision task. By combining behavioral measures, hierarchical drift-diffusion modeling, and event-related potential analyses, we aimed to characterize how expertise-related advantages unfold across different levels of sensory evidence and to identify the neural and computational mechanisms underlying these effects.

## 2. Materials and Methods

### 2.1. Participants

A total of 60 participants were initially recruited from Shanghai University of Sport (Shanghai, China) for the study. All participants were right-handed, had normal or corrected-to-normal vision, and reported no history of neurological or psychiatric disorders. Based on their sports background, participants were assigned to either an open-skill athlete group or a non-athlete control group, with equal numbers recruited for each group at the outset.

During preprocessing, participants were excluded if (i) accuracy was below 70% in the high-visibility conditions (levels 6–8), indicating insufficient task engagement, or (ii) more than 40% of EEG epochs were rejected due to artifacts. In addition, participants with fewer than 50% valid behavioral trials across the task were excluded.

All participants were university students aged between 18 and 24 years. The athlete group included participants from soccer (n = 8), basketball (n = 4), handball (n = 7), table tennis (n = 2), and Sanda (n = 4), with 11 males and 14 females. The control group included 12 males and 11 females. Written informed consent was obtained prior to the experiment. The experimental procedures were approved by the local ethics committee [18], and participants received monetary compensation for their participation. A participant flow diagram detailing recruitment, exclusions (with reasons), and the final analyzed sample is provided in Supplementary Figure S1.

### 2.2. Task and Stimuli

Participants completed a Gabor grating orientation discrimination task. Each trial began with the presentation of a fixation cross (“+”) at the center of the screen for a randomly jittered duration between 400 and 600 ms, in order to reduce temporal expectancy effects. A Gabor grating stimulus was then presented at the center of the screen for 17 ms. The grating was tilted either 30° to the left or 30° to the right.

Stimulus visibility was manipulated by applying a fixed opacity (alpha) value to the Gabor stimulus (α = 0.02, 0.04, 0.06, 0.08, 0.10, 0.12, 0.14, 0.16), defined a priori and applied uniformly to all participants. No individual staircase calibration was performed. Accordingly, ‘near-threshold’ refers to intermediate alpha levels defined at the group level, rather than individually calibrated thresholds. Following stimulus presentation, participants were required to indicate the orientation of the grating by pressing a corresponding button within a 3000 ms response window.

In Experiment 1a, participants were instructed to judge the orientation of the grating as quickly and accurately as possible (left: “f” key; right: “j” key). Each visibility level comprised 60 trials with equal numbers of left- and right-oriented stimuli. The experiment consisted of six blocks, each containing 80 trials, with all eight visibility levels presented 10 times per block in a randomized order. A total of 480 formal trials were completed. No feedback was provided during the formal experiment. Prior to the formal task, participants completed 30 practice trials with accuracy feedback.

**Figure 1:**
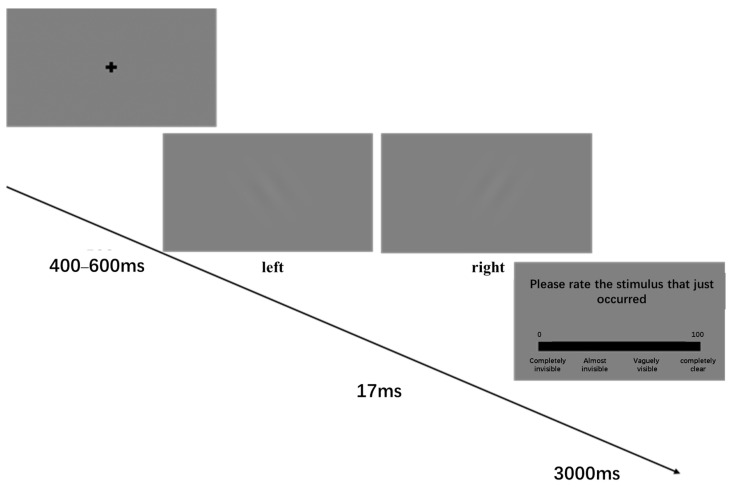
Experimental procedure of Experiment 1a.

After the orientation judgment, participants rated the subjective visibility of the stimulus using a continuous scale ranging from 1 (“completely invisible”) to 100 (“completely clear”). The response window for the visibility rating was also limited to 3000 ms (Figure 1). This two-stage response design allowed the collection of both objective behavioral performance and subjective awareness measures.

All experimental conditions were presented in a randomized order. Prior to the formal experiment, participants completed a practice session to familiarize themselves with the task procedure, response mapping, and visibility rating scale.

Experiment 1b employed a passive viewing (observation) task designed to characterize ERP responses as a function of stimulus visibility without requiring an overt behavioral response. Participants were instructed to maintain central fixation and to attentively observe each stimulus; no keypress or explicit report was required during the task.

Stimuli were identical to those in Experiment 1a. Visibility was manipulated by applying fixed stimulus opacity (alpha) values (α = 0.02, 0.04, 0.06, 0.08, 0.10, 0.12, 0.14, and 0.16), defined a priori and applied uniformly across participants. Each trial began with a fixation cross presented for 400–600 ms, followed by the stimulus presented for 17 ms, and an inter-trial interval of 500 ms.

The formal task consisted of 6 blocks with 80 trials per block (480 trials in total). Within each block, all eight visibility levels were presented 10 times in a randomized order. Short breaks were provided between blocks to reduce fatigue and maintain attention. Participants completed a brief practice session to familiarize themselves with the task instructions before the formal recording.

### 2.3. EEG Recording and Preprocessing

Continuous EEG data were recorded using a 64-channel ActiChamp system with a sampling rate of 1000 Hz. During acquisition, signals were referenced online to the CPz electrode. Offline preprocessing was performed in MATLAB (R2019a; MathWorks Inc., Natick, MA, USA) using the EEGLAB toolbox (v2023.0; Swartz Center for Computational Neuroscience, University of California San Diego, La Jolla, CA, USA) [19,20].

First, the data were re-referenced to the common average reference, with the FCz electrode restored during this step. A 50 Hz notch filter was applied to remove line noise, followed by a band-pass filter from 0.1 to 30 Hz with a slope of 24 dB/octave.

Ocular and muscular artifacts were identified and removed using independent component analysis (ICA). ICA components were classified using the ICLabel algorithm implemented in EEGLAB. Components were flagged for rejection if they exceeded the following probability thresholds: Eye > 90%, Muscle > 85%, Heart > 90%, and Line Noise > 90%. The final decision to remove components was made in combination with visual inspection of component scalp topographies, time courses, and power spectra, following standard EEGLAB recommendations [21].

The continuous EEG data were then segmented into epochs ranging from −200 to 1000 ms relative to stimulus onset. Baseline correction was applied using the −200 to 0 ms pre-stimulus interval. Epochs with voltage fluctuations exceeding ±80 μV were rejected as artifacts.

For each participant, artifact-free epochs were averaged separately for each of the eight stimulus visibility levels to obtain event-related potentials for subsequent analyses.

### 2.4. ERP Component Definition and Measurement

Based on the grand-average waveforms and prior literature on visual awareness and perceptual decision-making, three ERP components were selected for analysis: P2, N2, and P3.

The P2 component was defined as the mean amplitude within the 100–200 ms post-stimulus interval and is commonly associated with early perceptual processing. The N2 component was defined as the mean amplitude within the 180–280 ms interval, which has been linked to perceptual conflict and uncertainty processing [22,23]. The P3 component was defined as the mean amplitude within the 300–500 ms interval and is widely considered to reflect evidence accumulation and conscious access processes.

To reduce the number of statistical comparisons and improve robustness, electrodes were grouped into predefined regions of interest (ROIs) according to the international 10–20 system. The occipital ROI (O) included electrodes O1, Oz, and O2; the parieto-occipital ROI (PO) included PO1, POz, and PO2; the parietal ROI (P) included P1, Pz, and P2; and the central ROI (C) included C1, Cz, and C2. For each component, mean amplitudes were computed by averaging across electrodes within each ROI.

### 2.5. Statistical Analysis

All statistical analyses were conducted in the R environment (R version 4.5.1; R Foundation for Statistical Computing, Vienna, Austria). The significance level was set at α = 0.05, and all tests were two-tailed.

#### 2.5.1. Behavioral Data Analysis

Reaction time analyses included all valid trials, regardless of response accuracy, in order to capture the full dynamics of perceptual decision-making across visibility levels. Prior to analysis, trials with reaction times shorter than 150 ms or longer than 3000 ms were excluded. Reaction times were analyzed using a 2 (Group: athlete vs. non-athlete) × 8 (Stimulus visibility) repeated-measures analysis of variance.

Accuracy data, due to their binary nature, were analyzed using generalized linear mixed-effects models with a logit link function. Group and stimulus visibility were entered as fixed effects, and random effects were included at the participant level.

#### 2.5.2. ERP Data Analysis

ERP analyses were conducted separately for the P2, N2, and P3 components. Stimulus visibility and ROI were treated as within-subject factors, and group was treated as a between-subject factor in mixed-design analyses of variance.

Sphericity was assessed using Mauchly’s test. When the sphericity assumption was violated, Greenhouse–Geisser corrections were applied to the degrees of freedom, and corrected *p*-values were reported [24]. Effect sizes were reported as partial eta squared (η^2^p). Post hoc comparisons and simple effects analyses were conducted using Bonferroni correction when appropriate. Given the number of correlation tests across ROIs, components, visibility levels, and behavioral measures, *p*-values were controlled using the false discovery rate (FDR) procedure (q < 0.05). We report both uncorrected *p*-values and FDR-adjusted q-values. These correlation analyses are intended as exploratory, and we focus interpretation on the overall pattern (e.g., directionality and regional consistency) rather than any single *p*-value.

#### 2.5.3. Drift-Diffusion Modeling

To further characterize the computational mechanisms underlying perceptual decision-making, hierarchical drift-diffusion modeling was applied to the behavioral data [16,25]. In contrast to the behavioral analyses, a more conservative reaction time window (250–3000 ms) was used for model fitting to improve parameter stability. The model focused on key parameters including drift rate, non-decision time, boundary separation, and starting point bias, and examined how these parameters varied as a function of group and stimulus visibility.

This lower cutoff was chosen based on the empirical RT distribution to improve parameter stability. As a robustness check, we refit simplified HDDMs using alternative lower cutoffs (150 ms and 200 ms) and obtained qualitatively consistent parameter patterns; trials faster than 250 ms constituted only 0.3% of all trials.

## 3. Results

### 3.1. Behavioral Results: Accuracy

To appropriately model the binary nature of accuracy data while accounting for between-subject variability, accuracy was analyzed using a generalized linear mixed-effects model (GLMM) with a logit link function [26,27]. Group (athletes vs. non-athletes) and stimulus visibility (eight levels) were included as fixed effects, with random effects specified at the participant level.

The main effect of group was not significant, b = −0.15, SE = 0.12, z = −1.20, *p* = 0.23, indicating no overall difference in mean accuracy between athletes and non-athletes. In contrast, stimulus visibility was a strong predictor of accuracy, with performance increasing monotonically as visibility increased. All visibility levels showed significantly higher accuracy compared with the lowest visibility condition (all *p*s < 0.001).

**Figure 2:**
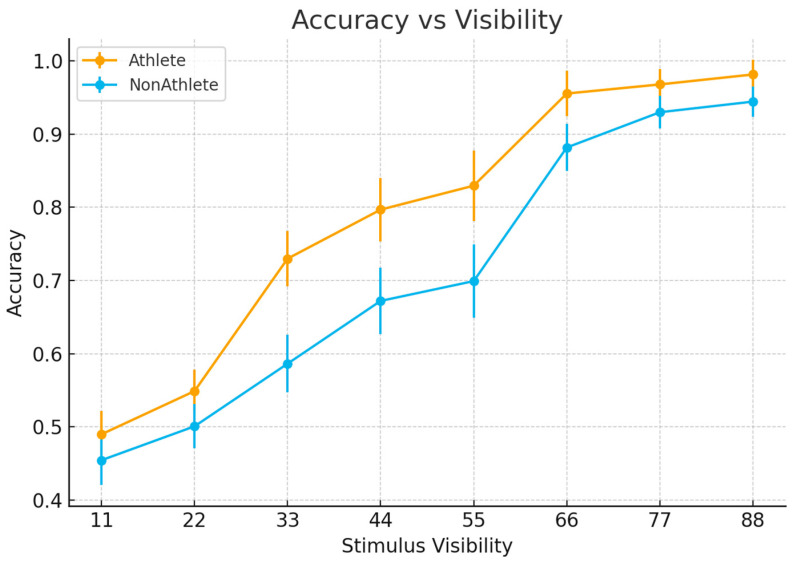
Accuracy as a function of stimulus visibility.

Critically, a significant Group × Visibility interaction was observed. Post hoc comparisons revealed that, at mid-to-high visibility levels, non-athletes exhibited significantly lower accuracy than athletes. The estimated interaction effects ranged from −0.51 to −0.97 on the log-odds scale, and all corresponding comparisons reached significance (*p*s < 0.001). No significant group differences were observed at the lowest visibility levels (Figure 2).

As a complementary analysis, a 2 (Group) × 8 (Visibility) repeated-measures ANOVA was conducted on accuracy data and yielded converging results, showing a significant main effect of visibility and a significant Group × Visibility interaction (all *p*s < 0.001, Greenhouse–Geisser corrected). The consistency between GLMM and ANOVA results further supports the robustness of the observed interaction.

### 3.2. Behavioral Results: Reaction Time

Reaction time (RT) data were analyzed using a 2 (Group: athletes vs. non-athletes) × 8 (Stimulus Visibility) repeated-measures ANOVA.

**Figure 3:**
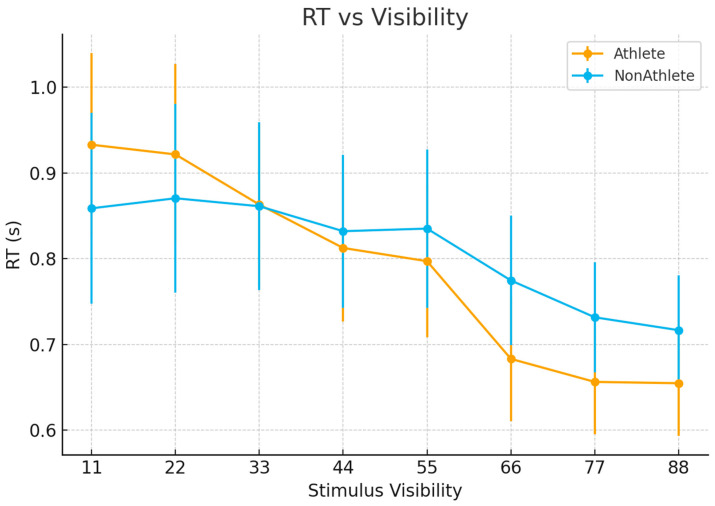
Reaction time as a function of stimulus visibility.

The main effect of group was not significant, F(1, 46) = 0.12, *p* = 0.73, indicating no overall difference in mean response speed between the two groups, as shown in Figure 3. In contrast, the main effect of stimulus visibility was significant, F(7, 322) = 47.92, *p* < 0.001 (Greenhouse–Geisser corrected), reflecting a systematic decrease in RT as stimulus visibility increased.

Importantly, the Group × Visibility interaction reached significance, F(7, 322) = 5.64, *p* = 0.008 (Greenhouse–Geisser corrected). Follow-up simple effects analyses showed that RT differences between groups were not significant at low to intermediate visibility levels (levels 1–5). At higher visibility levels (levels 6–7), athletes tended to respond faster than non-athletes, with this difference reaching marginal significance (*p*s ≈ 0.08).

### 3.3. Subjective Visibility Ratings

Subjective visibility ratings were analyzed using a 2 (Group) × 8 (Stimulus Visibility) repeated-measures ANOVA.

**Figure 4:**
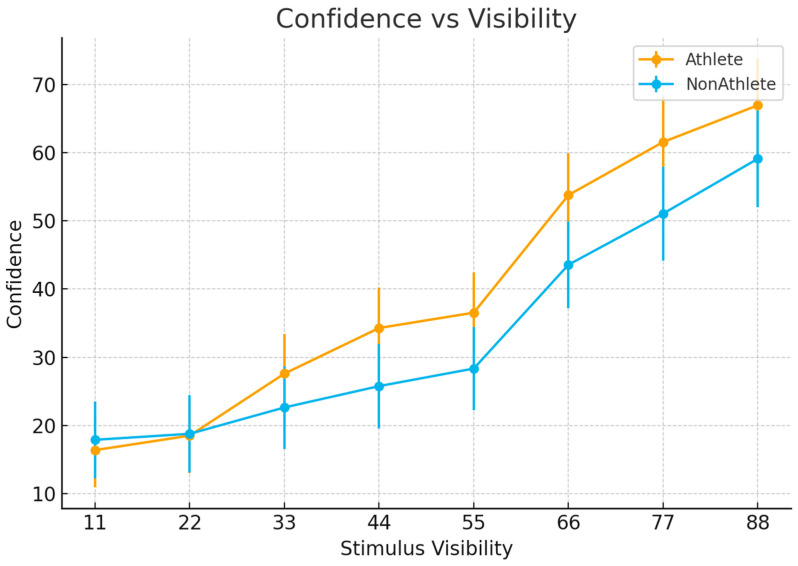
Subjective visibility ratings as a function of stimulus visibility.

The main effect of group was not significant, F(1, 46) = 2.77, *p* = 0.10, η^2^p = 0.057, indicating comparable overall subjective visibility ratings between athletes and non-athletes. In contrast, the main effect of stimulus visibility was highly significant, F(7, 322) = 194.40, *p* < 0.001 (Greenhouse–Geisser corrected), η^2^p = 0.809, showing that subjective visibility ratings increased strongly with increasing stimulus visibility (Figure 4).

Crucially, a significant Group × Visibility interaction was observed, F(7, 322) = 3.41, *p* = 0.047 (Greenhouse–Geisser corrected), η^2^p = 0.069. Post hoc analyses revealed that, at mid-to-high visibility levels (levels 3–8), athletes reported significantly higher subjective visibility than non-athletes, whereas no group differences were observed at the lowest visibility levels (levels 1–2).

### 3.4. Drift-Diffusion Modeling Results

To further elucidate the latent decision-making mechanisms underlying the observed behavioral differences, a hierarchical drift-diffusion model (HDDM) was fitted to the reaction time and accuracy data. The model simultaneously estimated core parameters of the decision process, including drift rate (v), non-decision time (ndt), boundary separation (a), and starting point (z).

**Figure 5:**
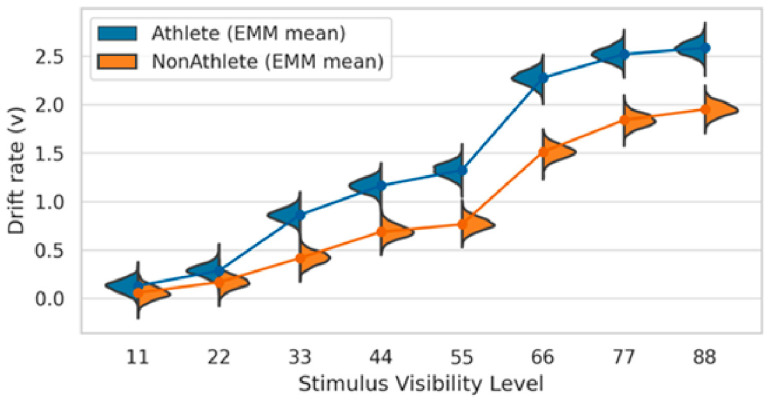
Group comparison of drift rate (v) as a function of stimulus visibility.

Drift Rate (v). Drift rate exhibited a pronounced effect of stimulus visibility, increasing monotonically across the eight visibility levels [16,28]. In the athlete group, drift rate increased from approximately 0.13 at the lowest visibility level to approximately 2.59 at the highest visibility level. A similar monotonic increase was observed in the non-athlete group, indicating that higher stimulus visibility systematically improved the quality and speed of evidence accumulation in both groups (Figure 5).

Crucially, a clear Group × Visibility interaction emerged in drift rate. At the lowest visibility levels (levels 1–2), no reliable group differences were observed, and the posterior credible intervals largely overlapped. However, starting from visibility level 3, athletes exhibited significantly higher drift rates than non-athletes. Across all mid-to-high visibility levels (levels 3–8), the 95% posterior credible intervals of the group differences consistently excluded zero (e.g., Δv = 0.45 at level 3; Δv = 0.76 at level 6; see Table 1). These group differences remained robust even at the highest visibility levels.

Taken together, these results indicate that, once sensory information reached a usable level, athletes accumulated task-relevant evidence more efficiently than non-athletes.

Drift rates increased monotonically with stimulus visibility in both groups. From intermediate visibility levels onward, athletes exhibited consistently higher drift rates than non-athletes, indicating more efficient evidence accumulation.

Non-decision Time (ndt). Non-decision time remained highly stable across all experimental conditions (overall mean M = 0.297 s, 95% credible interval = [0.279, 0.314]) and did not show systematic variation as a function of group or stimulus visibility. This suggests that athletes and non-athletes did not differ in early sensory encoding or motor execution processes.

Boundary Separation (a). Boundary separation estimates were highly consistent across conditions (a ≈ 1.59), with no significant effects of group or stimulus visibility. This pattern indicates that both groups adopted comparable levels of response caution, thereby ruling out speed–accuracy trade-offs or strategic response differences as an explanation for the observed behavioral effects.

Starting Point (z). Starting point estimates clustered around 0.47 across all conditions, with no reliable group differences. This suggests an absence of systematic response bias toward either decision boundary in both groups.

Overall, the drift-diffusion modeling results reveal a selective and coherent mechanism underlying the behavioral advantage observed in athletes. Group differences were confined to drift rate, indicating enhanced efficiency of evidence accumulation, whereas non-decision time, response caution, and starting point remained comparable between groups. These findings support the conclusion that the superior performance of athletes arises from more efficient transformation of sensory evidence into decision variables, rather than from differences in response strategy, motor execution, or bias.

### 3.5. Event-Related Potential Results

#### 3.5.1. Experiment 1a: Active Decision Task

**Figure 6:**
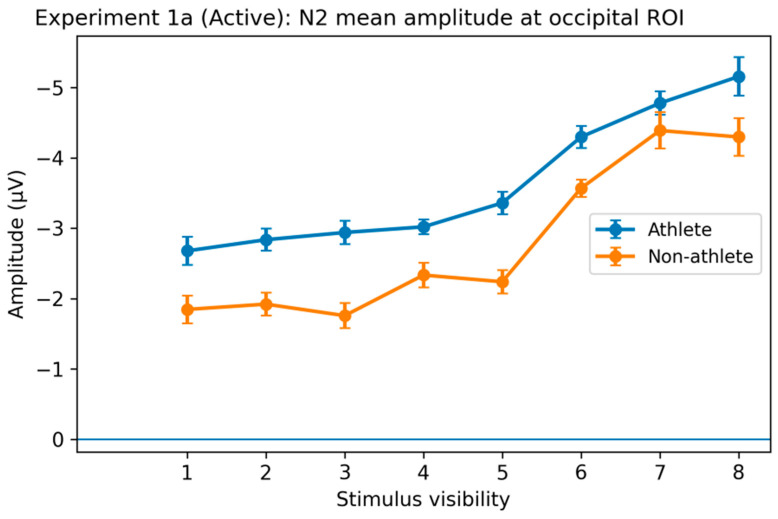
N2 component response to stimulus visibility in the active decision task of Experiment 1a. Average N2 amplitude (180–280 ms) in the occipital region (O: O1/Oz/O2) across different stimulus visibility levels. In both groups, N2 amplitude varied systematically with stimulus visibility, with significantly different responses evoked by higher visibility conditions (levels 6–8) compared to lower visibility conditions (levels 1–3). No stable main effect of group or group × visibility interaction was observed. Error bars represent within-subject standard error of the mean (SEM).

N2 Component. Mixed-design analyses of variance revealed a significant main effect of stimulus visibility on N2 amplitude in both the occipital (O) and parieto-occipital (PO) regions (O: F(3.47, 166.68) = 60.68, *p* < 0.001, η^2^p = 0.56; PO: F(3.47, 166.68) = 19.26, *p* < 0.001, η^2^p = 0.29), indicating that early visual processing was strongly modulated by stimulus strength. Post hoc comparisons showed that higher visibility levels (levels 6–8) elicited significantly different N2 responses compared to lower visibility levels (levels 1–3) in both groups (Figure 6).

No significant main effect of group or Group × Visibility interaction was observed (all *p*s > 0.05). Although isolated group differences were detected at specific visibility levels and regions, these effects lacked a consistent spatial distribution and did not form a systematic pattern across visibility levels. Overall, the N2 component primarily reflected visibility-dependent early sensory processing that was highly similar between athletes and non-athletes [22].

P2 Component. Significant main effects of stimulus visibility on P2 amplitude were observed across multiple regions, including central, fronto-central, occipital, and parieto-occipital areas (all *p*s < 0.001, η^2^p = 0.17–0.20), indicating that intermediate-stage perceptual processing was modulated by stimulus visibility.

**Figure 7:**
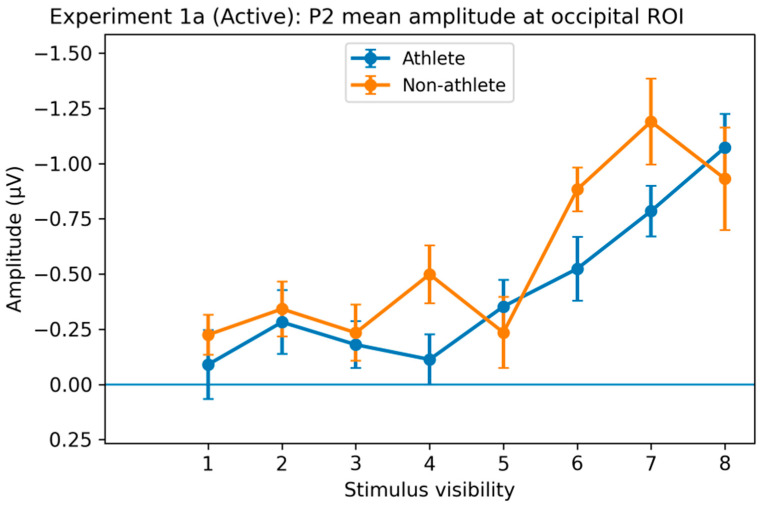
Stimulus visibility effects on the P2 component in the active decision task of Experiment 1a. Average P2 amplitude (100–200 ms) in the occipital region (O). All regions showed a significant main effect of stimulus visibility, reflecting modulation of intermediate-stage perceptual processing by stimulus intensity. Although group × visibility interactions were observed in some regions, their spatial distribution and direction were inconsistent and thus not interpreted as a primary finding. Error bars represent within-subject SEM.

In addition, significant Group × Visibility interactions were detected in selected regions, including the central region (F(5.61, 269.37) = 2.25, *p* = 0.043, η^2^p = 0.04) and the parietal region (F(4.69, 225.03) = 3.76, *p* = 0.003, η^2^p = 0.17). However, these interaction effects were region-specific and lacked a consistent scalp distribution or directional pattern. Given their limited spatial coherence, P2-related interaction effects are reported here for completeness but were not considered a primary focus of interpretation (Figure 7).

**Figure 8:**
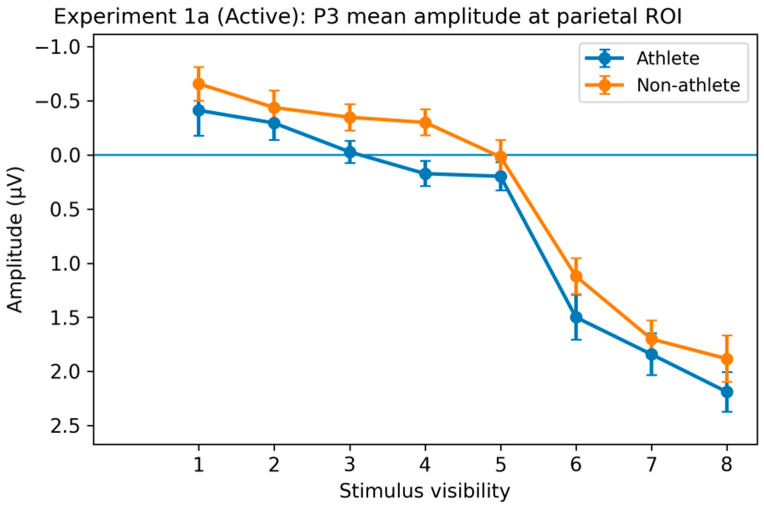
Group and visibility effects on the P3 component in the active decision task of Experiment 1a. Average P3 amplitude (300–500 ms) in the parietal region (P). P3 amplitude increased monotonically with stimulus visibility in both groups. Although the overall group × visibility interaction did not reach significance, post hoc analyses revealed that the athlete group showed significant P3 differentiation at intermediate visibility levels (levels 4–5), whereas significant differentiation in the non-athlete group primarily occurred at higher visibility levels (levels 6–8). Error bars represent within-subject SEM.

P3 Component. Significant main effects of stimulus visibility on P3 amplitude were observed in parietal, occipital, and fronto-central regions. As a representative example, the parietal region showed a robust visibility effect (F(2.80, 134.62) = 75.18, *p* < 0.001, η^2^p = 0.61), with comparable patterns observed across other regions of interest. In both groups, P3 amplitude increased monotonically with stimulus visibility, and higher visibility levels (6–8) elicited significantly larger amplitudes than lower visibility levels (1–3) (Figure 8).

Although the overall Group × Visibility interaction did not reach significance (all *p*s > 0.05), post hoc analyses revealed reliable group differences at specific visibility ranges. These differences were primarily observed at mid-to-high visibility levels (approximately levels 4–8) across multiple posterior and fronto-central regions, whereas no significant group differences were detected at the lowest visibility levels (levels 1–2).

Further within-group comparisons indicated distinct patterns of P3 differentiation across visibility levels. In the athlete group, P3 amplitudes at intermediate visibility levels (levels 4–5) were already significantly larger than those at the lowest visibility levels (levels 1–3; all *p*s < 0.05), with robust differences persisting at higher visibility levels (levels 6–8; all *p*s < 0.01). In contrast, in the non-athlete group, significant within-group differentiation emerged primarily at higher visibility levels (levels 6–8), whereas contrasts involving intermediate visibility levels were weaker or did not reach significance.

Overall, the ERP results from Experiment 1a reveal a clear dissociation across processing stages: early sensory processing indexed by the N2 component was largely comparable between groups, whereas later-stage processing indexed by the P3 component was highly sensitive to stimulus visibility and exhibited group-related differences in the threshold at which neural differentiation emerged.

#### 3.5.2. Experiment 1b: Passive Viewing Task

**Figure 9:**
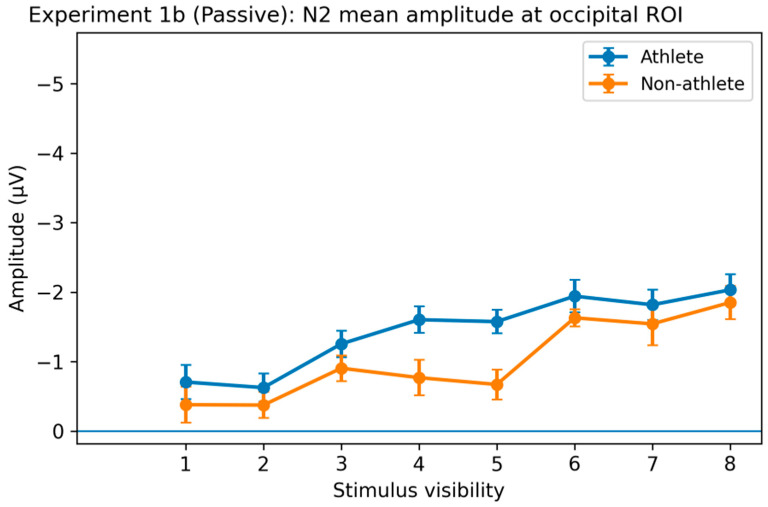
Stimulus visibility effects on the N2 component in the passive viewing task of Experiment 1b. Average N2 amplitude (180–280 ms) in the occipital region (O). Both regions showed a significant main effect of stimulus visibility, with lower visibility conditions (levels 1–2) eliciting smaller N2 amplitudes compared to higher visibility conditions (levels 6–8). A significant group × visibility interaction was observed in the parieto-occipital region, reflecting a more pronounced modulation of N2 amplitude by visibility in the athlete group. Error bars represent within-subject SEM.

N2 Component. In the passive viewing task, significant main effects of stimulus visibility on N2 amplitude were observed in both the occipital (O) and parieto-occipital (PO) regions (both *p*s < 0.001), indicating that early neural responses were systematically modulated by stimulus visibility even in the absence of explicit decision requirements. Post hoc analyses showed that low-visibility stimuli (levels 1–2) elicited significantly smaller N2 amplitudes than high-visibility stimuli (levels 6–8) in both regions (Bonferroni-corrected *p*s < 0.05), whereas differences among intermediate visibility levels were not significant (Figure 9).

Importantly, a significant Group × Visibility interaction was observed in the parieto-occipital region (F(4.67, 224.11) = 2.51, *p* = 0.034, η^2^p = 0.05). Follow-up analyses indicated that N2 amplitudes increased more strongly with visibility in the athlete group than in the non-athlete group. No significant main effect of group was observed in either region.

**Figure 10:**
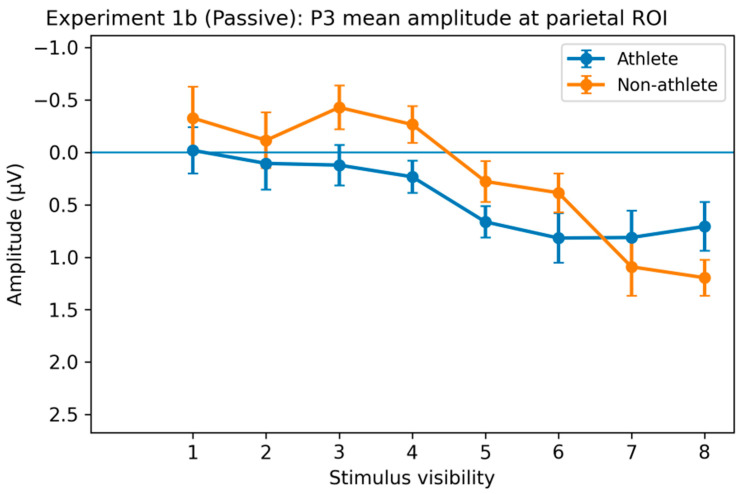
Stimulus visibility effects on the P3 component in the passive viewing task of Experiment 1b. Average P3 amplitude (300–500 ms) in the parietal region (P). A significant main effect of stimulus visibility was found for P3 amplitude, with higher visibility conditions (levels 6–8) eliciting larger amplitudes than lower visibility conditions (levels 1–2). No significant main effect of group or group × visibility interaction was observed. Error bars represent within-subject SEM.

P3 Component. Analysis of the parietal region revealed a significant main effect of stimulus visibility on P3 amplitude (F(4.14, 198.91) = 9.20, *p* < 0.001, η^2^p = 0.16). Post hoc comparisons showed that high-visibility stimuli (levels 6–8) elicited significantly larger P3 amplitudes than low-visibility stimuli (levels 1–2; Bonferroni-corrected *p*s < 0.05). No significant main effect of group or Group × Visibility interaction was observed (Figure 10).

### 3.6. ERP–Behavior Correlation Analyses

Correlation analyses between ERP amplitudes and behavioral measures were conducted to explore potential coupling patterns. Given the number of tests across ROIs and components, results are interpreted with caution, and emphasis is placed on effect direction and consistency rather than on the number of significant findings.

#### 3.6.1. Overall Correlations Collapsing Across Visibility Levels

To examine stable individual differences that were not tied to specific stimulus visibility levels, correlations between ERP component amplitudes and behavioral measures were computed after collapsing across all visibility conditions. Correlation analyses were conducted separately for the athlete and non-athlete groups [9,29].

Clear group differences emerged. Compared with the non-athlete group, the athlete group exhibited a greater number of significant ERP–behavior correlations, with stronger effect sizes and a broader spatial distribution. Across behavioral measures (accuracy, reaction time, and subjective visibility), significant correlations were primarily concentrated in the P3 component, whereas N2 and P2 components showed only sparse and inconsistent associations in both groups.

In the athlete group, P3 amplitude showed significant and region-specific correlations with reaction time. Specifically, P3 amplitudes in central and fronto-central regions were positively correlated with reaction time, such that larger P3 amplitudes were associated with slower responses (e.g., Cz: r = 0.48, *p* = 0.015; Fz: r = 0.53, *p* = 0.006), which remained at a trend level after FDR correction (Cz: q = 0.090; Fz: q = 0.0584). No comparable association was observed in the control group. In contrast, occipital P3 amplitude was negatively associated with reaction time in athletes (r = −0.46, *p* = 0.022), with the effect remaining marginal after FDR correction (q = 0.099), whereas no corresponding effect was found in controls, indicating that larger posterior P3 amplitudes were associated with faster responses.

No stable or significant ERP–reaction time correlations were observed in any region in the non-athlete group.

Across regions of interest and behavioral measures, correlation coefficients in the athlete group generally fell within the moderate range (|r| ≈ 0.30–0.50), indicating a reliable coupling between late-stage neural activity and behavioral efficiency at the individual level. Notably, these correlations were not confined to a single region but spanned multiple posterior ROIs, suggesting a relatively systematic neural–behavioral linkage.

By contrast, the non-athlete group exhibited fewer significant ERP–behavior correlations, with a more restricted spatial distribution, primarily involving parietal or centro-parietal regions. Although the direction of correlations was broadly consistent with that observed in athletes (i.e., larger P3 amplitudes associated with higher accuracy and subjective visibility and shorter reaction times), their overall strength was weaker, typically ranging from |r| ≈ 0.20–0.35.

These associations did not meet the conventional FDR threshold (q < 0.05) and are therefore interpreted as exploratory and supportive of the main findings rather than as confirmatory evidence.

**Figure 11:**
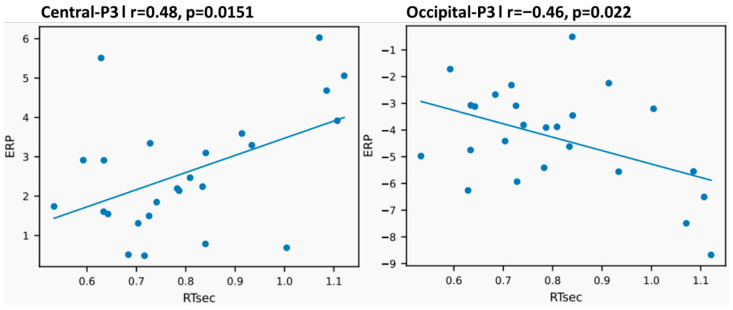
Examples of the correlation between P3 amplitude and reaction time in the athlete group (central area Cz and occipital area Oz). Scatter plots show the relationship between mean reaction time (RT, seconds) and P3 amplitude (μV) at the central electrode (Cz, **left**) and occipital electrode (Oz, **right**), collapsed across stimulus visibility levels. Each dot represents one participant. Solid lines indicate least-squares regression fits.

Representative examples of P3 amplitude–reaction time correlations in the athlete group (central site Cz and occipital site Oz) are shown in Figure 11. A summary of the number and effect size ranges of significant ERP–behavior correlations collapsed across visibility levels is provided in Table 2. Full correlation results are reported in the Supplementary Materials.

#### 3.6.2. Visibility-Stratified Correlation Analyses

To further explore how ERP–behavior relationships varied as a function of stimulus visibility, correlation analyses were conducted separately for each visibility level. Given the large number of tests across bins, ROIs, and behavioral measures, these analyses are interpreted as exploratory, with emphasis placed on overall patterns rather than on individual effects.

**Figure 12:**
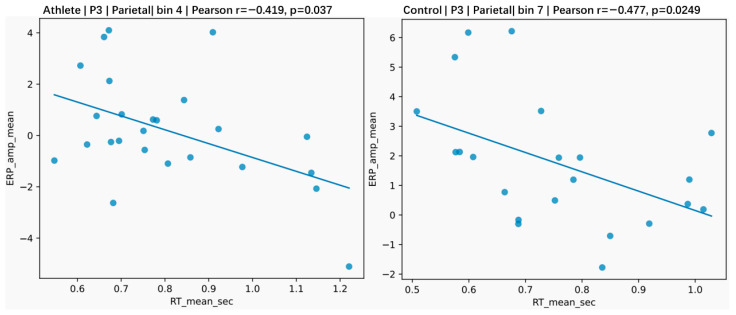
Representative examples of the association between P3 amplitude and reaction time at different visibility levels. The (**left**) panel illustrates an example from the athlete group at a medium visibility level (bin 4), and the (**right**) panel illustrates an example from the control group at a high visibility level (bin 7). These plots are shown for illustrative purposes to highlight visibility-dependent differences in ERP–behavior coupling patterns.

In the athlete group, ERP–behavior associations were already observable at low to intermediate levels of stimulus visibility (approximately bins 3–5), where P3 amplitude showed uncorrected associations with behavioral measures such as reaction time and accuracy (Figure 12). However, after controlling for multiple comparisons using FDR correction, these associations were reduced to trend-level effects and did not meet the conventional significance threshold (q < 0.05).

As stimulus visibility increased further, ERP–behavior associations in the athlete group became more robust. At high visibility levels (approximately bins 6–8), a subset of P3–behavior correlations remained significant after FDR correction (q < 0.05), indicating a stable coupling between late-stage neural processing and behavioral performance when perceptual evidence was strong. These effects were observed across central and posterior ROIs.

In contrast, the non-athlete group showed a markedly different pattern. ERP–behavior associations were weak or absent at low to intermediate visibility levels, and few associations remained significant after FDR correction even at high visibility levels. Overall, ERP–behavior coupling in non-athletes appeared less robust and more dependent on fully visible stimuli.

Taken together, the visibility-stratified analyses suggest that while early-stage trends in ERP–behavior coupling may already be present in athletes at lower visibility levels, statistically robust coupling emerges primarily at high visibility levels and is largely specific to the athlete group. These findings should be interpreted as exploratory and supportive of the main results.

## 4. Discussion

A consistent finding across all levels of analysis was the absence of reliable differences between athletes and non-athletes at the lowest levels of stimulus visibility. At these levels, the two groups showed highly similar performance across behavioral measures (accuracy and reaction time), computational indices (drift rate), and neural markers (N2 and P3 components) [1]. This result indicates that long-term sports training does not lead to a generalized enhancement of perceptual sensitivity or decision efficiency when sensory evidence is extremely limited and insufficient to support stable decision formation.

Importantly, this “null effect” constitutes a meaningful boundary condition for expertise-related advantages. Rather than reflecting a failure of expertise to manifest, it is more appropriately interpreted as indicating a minimum informational requirement for such advantages to emerge. Only when stimulus visibility exceeds a certain threshold can perceptual information be stably integrated and transformed into decision-relevant evidence [30,31], allowing potential individual differences to become observable at behavioral and neural levels. When perceptual evidence is severely constrained, the benefits conferred by systematic training appear insufficient to alter the overall output of the perception–decision cascade.

As stimulus visibility increased, group differences began to emerge in a coordinated manner across multiple analytical levels [3,17,32]. Behaviorally, athletes showed higher accuracy at intermediate to high visibility levels and exhibited a tendency toward faster responses at the highest visibility levels. Although reaction-time differences reached only marginal significance, their direction was consistent with the accuracy results, suggesting more efficient decision performance in athletes when perceptual information was of sufficient quality.

This behavioral pattern was further clarified at the computational level. Drift-diffusion modeling revealed no systematic group differences in non-decision time, response caution, or starting point bias, thereby ruling out explanations based on speed–accuracy trade-offs or strategic response differences. Instead, group differences were selectively expressed in the drift-rate parameter [16,25], and these differences emerged reliably from intermediate visibility levels onward. This finding indicates that once perceptual information becomes usable, athletes are able to accumulate task-relevant evidence more efficiently.

Electrophysiological results further constrained the processing stage at which expertise exerts its influence. Early N2 components showed strong main effects of stimulus visibility in both experiments, indicating high sensitivity of early sensory processing to stimulus strength. However, in the active decision task, N2 did not show stable group differences, suggesting that sports expertise does not systematically alter early sensory encoding or early conflict processing [22]. In contrast, the later P3 component exhibited a pattern closely aligned with both behavioral and computational results. In Experiment 1a, although the overall group × visibility interaction did not reach significance, post hoc analyses clearly revealed group differences in the visibility level at which neural differentiation emerged: athletes showed significant P3 amplitude differentiation at intermediate visibility levels, whereas comparable differentiation in non-athletes primarily appeared at higher visibility levels. This earlier emergence of neural differentiation suggests that sports expertise does not enhance early sensory input but instead accelerates and amplifies later neural processes associated with evidence accumulation and conscious access [23,32,33]. This interpretation is consistent with recent models proposing that P3 amplitude reflects the accumulation of decision-related evidence rather than early sensory encoding [34].

This neural “threshold shift” closely mirrors the drift-rate results and further supports the conclusion that expertise primarily affects later stages of evidence integration and decision formation rather than early visual processing. Notably, even at the level of P3, no group differences were observed under fully subliminal conditions, reinforcing the notion that expertise-related advantages depend on a minimum level of perceptual clarity.

The comparison between active decision and passive viewing tasks provides critical additional support for this interpretation. Although stimulus visibility robustly modulated N2 and P3 components in the passive viewing condition, indicating a degree of task-independent sensitivity to sensory evidence, group-related differences were substantially reduced and largely confined to early posterior regions. In contrast, when participants were required to actively evaluate stimuli and translate perceptual information into explicit behavioral responses, athletes’ advantages became more pronounced and systematic at both the computational level (drift rate) and the late neural level (earlier P3 differentiation). This contrast indicates that expertise-related advantages are not automatically triggered by stimulus input but depend on task contexts that require active integration, weighting, and utilization of perceptual evidence for decision output [4,32]. In other words, sports training appears to shape not passive sensory amplification but a capacity to use available perceptual evidence more efficiently when decision demands are present.

Finally, ERP–behavior correlation analyses provided key cross-level evidence for these conclusions. Athletes exhibited stronger, more stable, and earlier-emerging coupling between late neural activity and behavioral performance [29], with this coupling primarily centered on the P3 component associated with evidence accumulation and decision formation [35]. Recent studies have similarly reported that variability in late ERP components is predictive of individual differences in response speed and decision-related performance, supporting the functional link between P3 dynamics and behavioral output [36]. Notably, in the athlete group, P3 amplitude was already systematically related to reaction time, accuracy, and subjective visibility at intermediate visibility levels, whereas comparable coupling in non-athletes only emerged at high visibility levels. Although the P3–reaction time associations in athletes did not survive strict correction for multiple comparisons, their consistent directionality and absence in the control group suggest a potential link between late-stage neural processing and behavioral response speed in experts. These results should be interpreted as exploratory.

These results indicate that sports expertise not only shifts group-level averages in decision efficiency but also reshapes the coordination between neural processing and behavioral output at the individual level. Athletes’ advantages are therefore not limited to being “faster” or “more accurate,” but rather lie in how late neural processes are more tightly and efficiently linked to behavior. This cross-level coupling pattern provides important insight into how training in dynamic and uncertain environments optimizes perceptual–decision systems and further supports the conclusion that sports expertise primarily enhances the efficiency with which perceptual evidence is transformed into decision variables, rather than enhancing early sensory input or altering response strategies.

## 5. Conclusions

The present study investigated how open-skill sports expertise modulates perceptual decision-making across graded levels of stimulus visibility by integrating behavioral measures, hierarchical drift–diffusion modeling, and event-related potentials. Across all analytical levels, no reliable group differences were observed under fully subliminal conditions, indicating that sports expertise does not confer a generalized enhancement of unconscious sensory processing. Instead, group differences emerged selectively at near-threshold and suprathreshold visibility levels.

At the behavioral and computational levels, athletes showed higher accuracy and increased drift rates once sensory evidence reached a usable level, suggesting more efficient evidence accumulation rather than altered response strategies. At the neural level, early sensory processing indexed by N2 was similarly modulated by stimulus visibility in both groups, whereas late-stage processing indexed by the P3 component differentiated the groups. Athletes exhibited earlier and more robust P3 differentiation across visibility levels, along with stronger and more structured P3–behavior coupling.

Together, these findings indicate that open-skill sports expertise primarily optimizes the transformation of perceptual evidence into decision variables under conditions of uncertainty. Rather than enhancing early sensory input, expertise appears to refine late-stage neural computations that support efficient perceptual decisions near the threshold of awareness.

## Figures and Tables

**Table 1:** Group differences in drift rate (v) across stimulus visibility levels (posterior estimates and 95% credible intervals).

Contrast	Visibility	Estimate	Lower HPD	Upper HPD
Athlete–Non-athlete	1	0.07	−0.11	0.26
Athlete–Non-athlete	2	0.12	−0.06	0.30
Athlete–Non-athlete	3	0.45	0.26	0.63
Athlete–Non-athlete	4	0.48	0.31	0.67
Athlete–Non-athlete	5	0.56	0.37	0.73
Athlete–Non-athlete	6	0.77	0.57	0.95
Athlete–Non-athlete	7	0.68	0.48	0.87
Athlete–Non-athlete	8	0.63	0.42	0.81

**Table 2:** Summary of the Number and Effect Range of ERP-Behavior Correlations Meeting Uncorrected *p* < 0.05 under the Condition of Merging across Visibility Levels.

Group	ERP Component	Behavioral Measure	Number of Significant Correlations	r Range
**Athlete**	P3	Accuracy	8	0.32–0.51
**Athlete**	P3	Reaction time	7	−0.46–0.53
**Athlete**	P3	subjective visibility	9	0.34–0.53
**Athlete**	N2/P2	All	2	0.22–0.28
**Control**	P3	Accuracy	3	0.24–0.33
**Control**	P3	Reaction time	2	−0.26–−0.31
**Control**	P3	subjective visibility	3	0.25–0.35
**Control**	N2/P2	All	1	0.25

## Data Availability

The data presented in this study are available upon request to the corresponding author. The data are not publicly available due to privacy reasons.

## Supplementary Materials

The following supporting information can be downloaded at: https://www.mdpi.com/article/10.3390/brainsci16020198/s1, Figure S1: Participant Flow Diagram; File S1: ERP–behavior correlation results with bin (Excel file); File S2: ERP–behavior correlation report without bin (Word file).

## Abbreviations

The following abbreviations are used in this manuscript:

DDM	Drift-Diffusion Model
EEG	Electroencephalography
ERP	Event-Related Potential
FC	Frontocentral
GLMM	Generalized Linear Mixed-Effects Model
HDDM	Hierarchical Drift-Diffusion Model
N2	Negative Component Peaking Around 200 ms
P2	Positive Component Peaking Around 200 ms
P3	Positive Component Peaking Around 300 ms
PO	Parieto-Occipital
ROI	Region of Interest
RT	Reaction Time
SEM	Standard Error of the Mean
