# Supplementary material for: Perceptual Decision Advantages in Open-Skill Athletes Emerge near the Threshold of Awareness: Behavioral, Computational, and Electrophysiological Evidence
Source: Brain Sci. 2026 Feb 7;16(2):198. doi: 10.3390/brainsci16020198 (PMC12939444; doi:10.3390/brainsci16020198)
Supplement: Supplementary file 1 [file brainsci-16-00198-s001.zip › File S2. erp_behavior_correlations_by_group_report_with_components (1).pdf]

ERP–Behavior Correlation Report (by group)

Dataset: erp_behavior_cor.xlsx (subject-level aggregation across all stimulus bins/levels).

Groups analyzed separately: Athlete vs NonAthlete.

Behavior variables: ACC (mean accuracy), RTsec (mean RT in seconds), Conf (mean confidence), conf_level_mean (mean confidence bin).

ERP variables: N2, P2, P3 amplitudes averaged within each ROI (OZ, POZ, PZ, CPZ, CZ, FZ).

Statistics: Pearson r (with BH-FDR correction within each Group×Behavior across 18 ROI×Component tests) and Spearman rho (uncorrected). Full results are in the accompanying Excel file.

Sample sizes

Athlete: 25 subjects

Control: 22 subjects

FDR-significant Pearson correlations (summary)

Group	Behavior	# significant (FDR)
Athlete	ACC	0
Athlete	Conf	0
Athlete	RTsec	0
Athlete	conf_level_mean	0
Control	ACC	0
Control	Conf	0
Control	RTsec	0
Control	conf_level_mean	0

Note: even if FDR yields 0, the raw correlations are still listed in Excel and visualized in the PDF.

Outputs

1) Excel (all correlations + subject-level data): erp_behavior_correlations_by_group_with_components.xlsx

2) PDF (8 pages; each page shows all 18 ERP scatterplots for one Group×Behavior): erp_behavior_scatter_by_group_allvars.pdf

3) PNG pages (same 8 pages as images): erp_behavior_scatter_by_group_pages_pngs.zip

Top correlations (absolute Pearson r, per Group×Behavior)

Athlete | ACC | Occipital (Oz/O1/O2)-P3: r=0.552, p=0.0042, FDR q=0.0757

Athlete | ACC | Parieto-occipital (POz/PO1/PO2)-P3: r=0.483, p=0.0145, FDR q=0.131

Athlete | ACC | Central (Cz)-P3: r=-0.433, p=0.0305, FDR q=0.183

Athlete | ACC | Frontal (Fz/F1/F2)-P3: r=-0.360, p=0.0771, FDR q=0.347

Athlete | ACC | Central (Cz)-P2: r=0.318, p=0.122, FDR q=0.368

Athlete | Conf | Parieto-occipital (POz/PO1/PO2)-P2: r=0.568, p=0.00308, FDR q=0.0555

Athlete | Conf | Occipital (Oz/O1/O2)-P2: r=0.515, p=0.00836, FDR q=0.0753

Athlete | Conf | Parietal (Pz/P1/P2)-P2: r=0.352, p=0.0847, FDR q=0.508

Athlete | Conf | Central (Cz)-P2: r=-0.303, p=0.141, FDR q=0.552

Athlete | Conf | Occipital (Oz/O1/O2)-N2: r=0.293, p=0.155, FDR q=0.552

Athlete | RTsec | Parietal (Pz/P1/P2)-N2: r=-0.530, p=0.00643, FDR q=0.0584

Athlete | RTsec | Frontal (Fz/F1/F2)-P3: r=0.530, p=0.00648, FDR q=0.0584

Athlete | RTsec | Central (Cz)-P3: r=0.480, p=0.0151, FDR q=0.0904

Athlete | RTsec | Occipital (Oz/O1/O2)-P3: r=-0.456, p=0.022, FDR q=0.0988

Athlete | RTsec | Parieto-occipital (POz/PO1/PO2)-P3: r=-0.421, p=0.0363, FDR q=0.131

Athlete | conf_level_mean | Parieto-occipital (POz/PO1/PO2)-P2: r=0.570, p=0.00291, FDR q=0.0524

Athlete | conf_level_mean | Occipital (Oz/O1/O2)-P2: r=0.519, p=0.00789, FDR q=0.071

Athlete | conf_level_mean | Parietal (Pz/P1/P2)-P2: r=0.346, p=0.0902, FDR q=0.488

Athlete | conf_level_mean | Occipital (Oz/O1/O2)-N2: r=0.300, p=0.145, FDR q=0.488

Athlete | conf_level_mean | Central (Cz)-P2: r=-0.290, p=0.159, FDR q=0.488

Control | ACC | Central (Cz)-N2: r=-0.238, p=0.286, FDR q=0.975

Control | ACC | Centro-parietal (CPz)-N2: r=-0.230, p=0.304, FDR q=0.975

Control | ACC | Parieto-occipital (POz/PO1/PO2)-N2: r=0.183, p=0.415, FDR q=0.975

Control | ACC | Occipital (Oz/O1/O2)-N2: r=0.171, p=0.446, FDR q=0.975

Control | ACC | Frontal (Fz/F1/F2)-P3: r=-0.160, p=0.477, FDR q=0.975

Control | Conf | Parieto-occipital (POz/PO1/PO2)-N2: r=0.526, p=0.012, FDR q=0.216

Control | Conf | Occipital (Oz/O1/O2)-N2: r=0.456, p=0.0329, FDR q=0.247

Control | Conf | Frontal (Fz/F1/F2)-P2: r=-0.417, p=0.0534, FDR q=0.247

Control | Conf | Frontal (Fz/F1/F2)-N2: r=-0.413, p=0.0561, FDR q=0.247

Control | Conf | Central (Cz)-N2: r=-0.383, p=0.0785, FDR q=0.247

Control | RTsec | Centro-parietal (CPz)-P3: r=-0.400, p=0.0654, FDR q=0.821

Control | RTsec | Centro-parietal (CPz)-P2: r=-0.340, p=0.122, FDR q=0.821

Control | RTsec | Parietal (Pz/P1/P2)-P3: r=-0.327, p=0.137, FDR q=0.821

Control | RTsec | Frontal (Fz/F1/F2)-P3: r=0.245, p=0.272, FDR q=0.864

Control | RTsec | Parieto-occipital (POz/PO1/PO2)-P2: r=-0.219, p=0.328, FDR q=0.864

Control | conf_level_mean | Parieto-occipital (POz/PO1/PO2)-N2: r=0.568, p=0.00582, FDR q=0.105

Control | conf_level_mean | Occipital (Oz/O1/O2)-N2: r=0.513, p=0.0146, FDR q=0.131

Control | conf_level_mean | Central (Cz)-N2: r=-0.468, p=0.0279, FDR q=0.153

Control | conf_level_mean | Frontal (Fz/F1/F2)-N2: r=-0.446, p=0.0375, FDR q=0.153

Control | conf_level_mean | Frontal (Fz/F1/F2)-P2: r=-0.436, p=0.0424, FDR q=0.153
