# Supplementary figures and images for: Perceptual Decision Advantages in Open-Skill Athletes Emerge near the Threshold of Awareness: Behavioral, Computational, and Electrophysiological Evidence
Source: Brain Sci. 2026 Feb 7;16(2):198. doi: 10.3390/brainsci16020198 (PMC12939444; doi:10.3390/brainsci16020198)

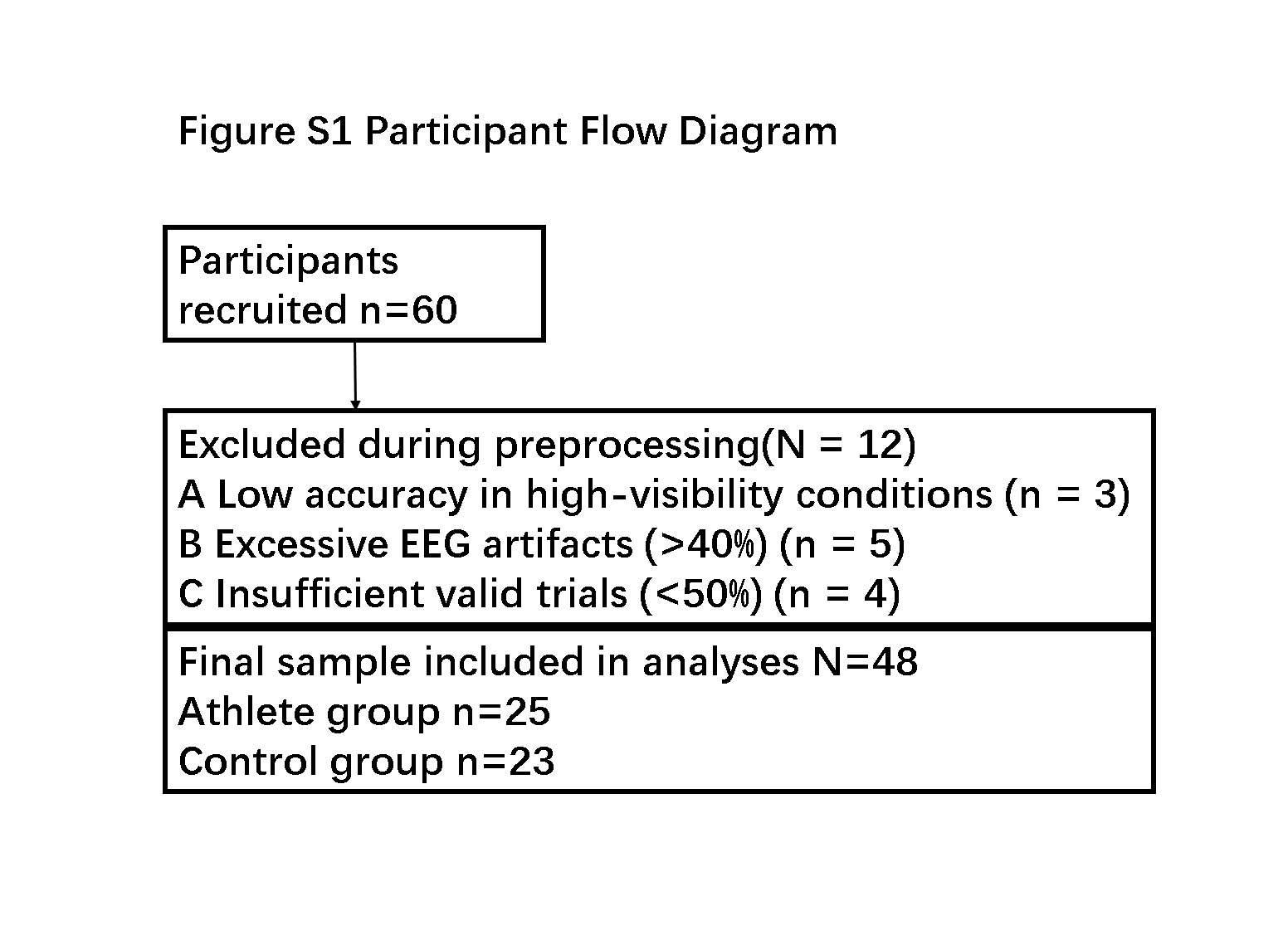

Supplement: Supplementary file 1 [file brainsci-16-00198-s001.zip › brainsci-4130190-supplementary.png]
